# Supplementary material for: Exercising D. melanogaster Modulates the Mitochondrial Proteome and Physiology. The Effect on Lifespan Depends upon Age and Sex
Source: Int J Mol Sci. 2021 Oct 27;22(21):11606. doi: 10.3390/ijms222111606 (PMC8583977; doi:10.3390/ijms222111606)
Supplement: Supplementary file 1 [file ijms-22-11606-s001.zip › ijms-1414494-supplementary.pdf]

Supplemental data

Supplementary Table S1 – Differences of mitochondrial protein expression in male flies that were exercised for one week (N=3). Statistically significant differences in protein expression (one-way ANOVA) were determined using SameSpots (Totallab). Protein identification was conducted using 2D-PAGE followed by MALDI-TOF/MS.

Spot no.	Protein identity	Anova (p)	Fold Change	Exercise related change
519	Arginine kinase OS=Drosophila melanogaster	0.003	2	Reduced
784	NADH-ubiquinone oxidoreductase 75 kDa subunit	0.004	1.5	Increased
773	Alcohol dehydrogenase	0.012	1.7	Reduced
791	Actin, larval muscle	0.024	2.7	Reduced
788	IP09655p (malate dehydrogenase)<br>Succinate-CoA ligase [ADP/GDP-forming] subunit alpha<br>ATP synthase subunit alpha	0.037	1.8	Reduced

Supplementary Table S2 - Difference in whole fly protein expression in response to one week of exercise (N=3). Statistically significant differences in protein expression (one-way ANOVA) were analysed using SameSpots, Totallab. Protein identification by MALDI-TOF/MS. F/M/g/se, female/male group/single exercised; F/M/g/sne, female/male group/single non-exercised.

Group	Spot no.	Protein Identity	Anova (p)	Fold change	Increased in
Fge vs Fse	349	Glycerol-3-phosphate dehydrogenase [NAD(+)], plus a single peptide match to Probable isocitrate dehydrogenase [NAD]	0.008	1.5	Fse
	358	Aconitate hydratase	0.016	1.2	Fge
Fge vs Fgne	351	Glycerol-3-phosphate dehydrogenase [NAD(+)]	0.032	1.7	Fgne
	363	Aconitate hydratase	0.047	1.5	Fge
	375	Vitellogenin-3	0.039	1.6	Fge
Mgne vs Fgne	472	Pyruvate dehydrogenase E1 component subunit beta	0.001	2.2	Mgne
	486	Fructose-bisphosphate aldolase	0.0009479	2.4	Fgne
Mge vs Mgne	487	Dihydrolipoyl dehydrogenase	0.0005776	1.7	Mgne

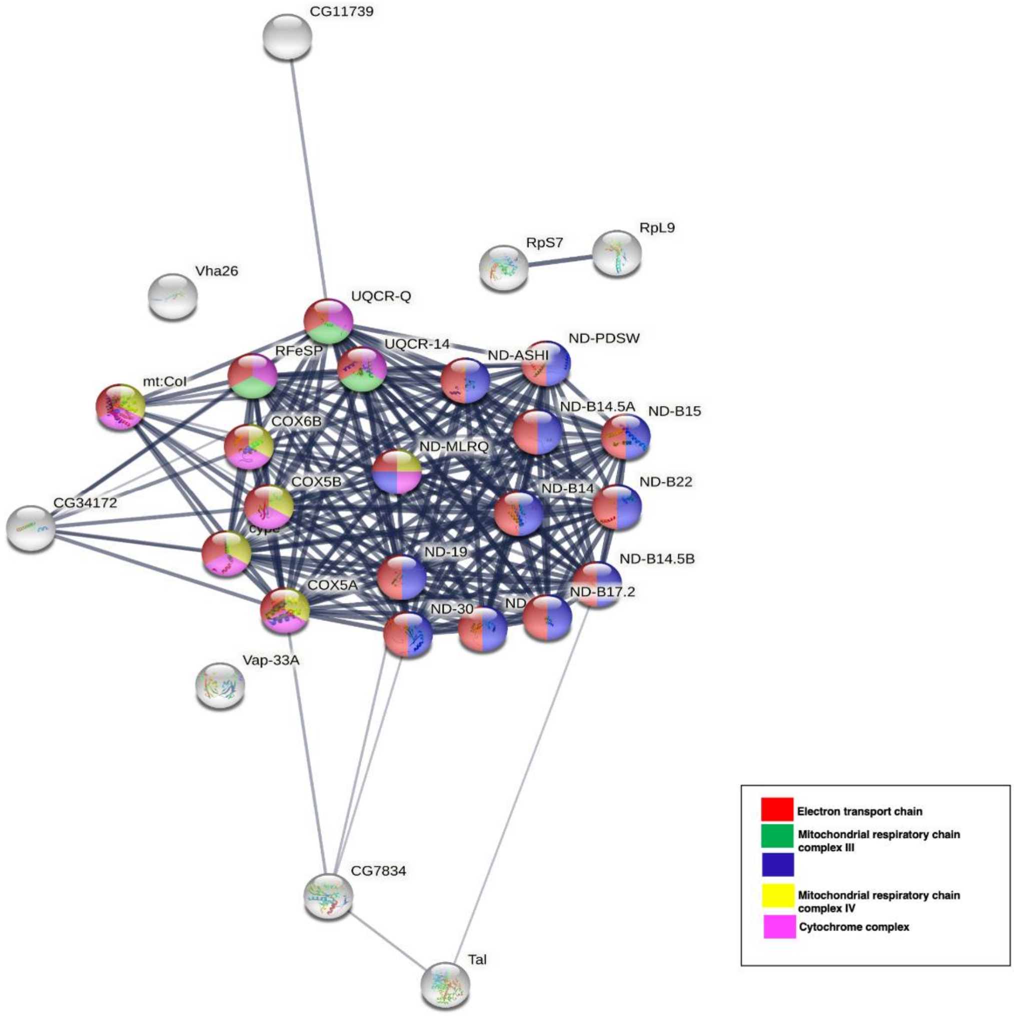
CG11739
RpS7
RpL9
Vha26
UQCR-Q
UQCR-14
RFeSP
ND-ASHI
ND-PDSW
mt:CoI
ND-B14.5A
ND-B15
COX6B
ND-MLRQ
COX5B
ND-B14
ND-B22
CG34172
ND-19
ND-B14.5B
COX5A
ND-B17.2
ND-30
Vap-33A
CG7834
Tal
Electron transport chain
Mitochondrial respiratory chain complex III
Mitochondrial respiratory chain complex IV
Cytochrome complex

Figure S1

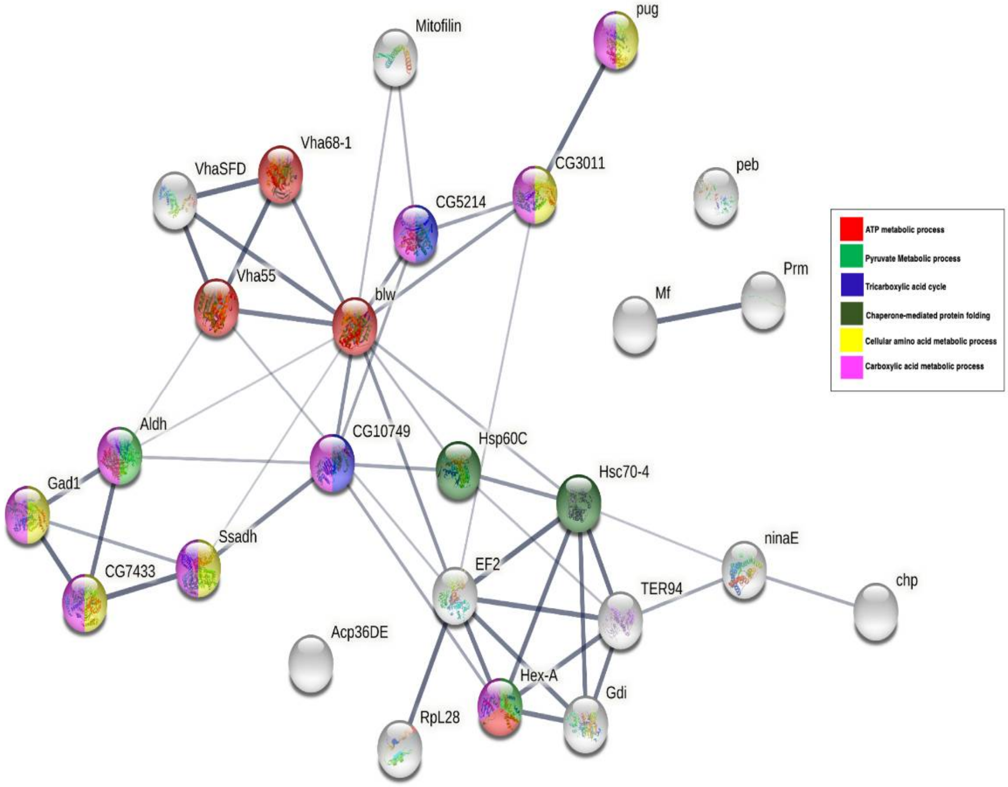
Mitofilin
pug
Vha68-1
VhaSFD
CG3011
peb
CG5214
Vha55
blw
Prm
Mf
ATP metabolic process
Pyruvate Metabolic process
Tricarboxylic acid cycle
Chaperone-mediated protein folding
Cellular amino acid metabolic process
Carboxylic acid metabolic process
Aldh
CG10749
Hsp60C
Hsc70-4
Gad1
Ssadh
CG7433
EF2
TER94
ninaE
chp
Acp36DE
Hex-A
Gdi
RpL28

Figure S2

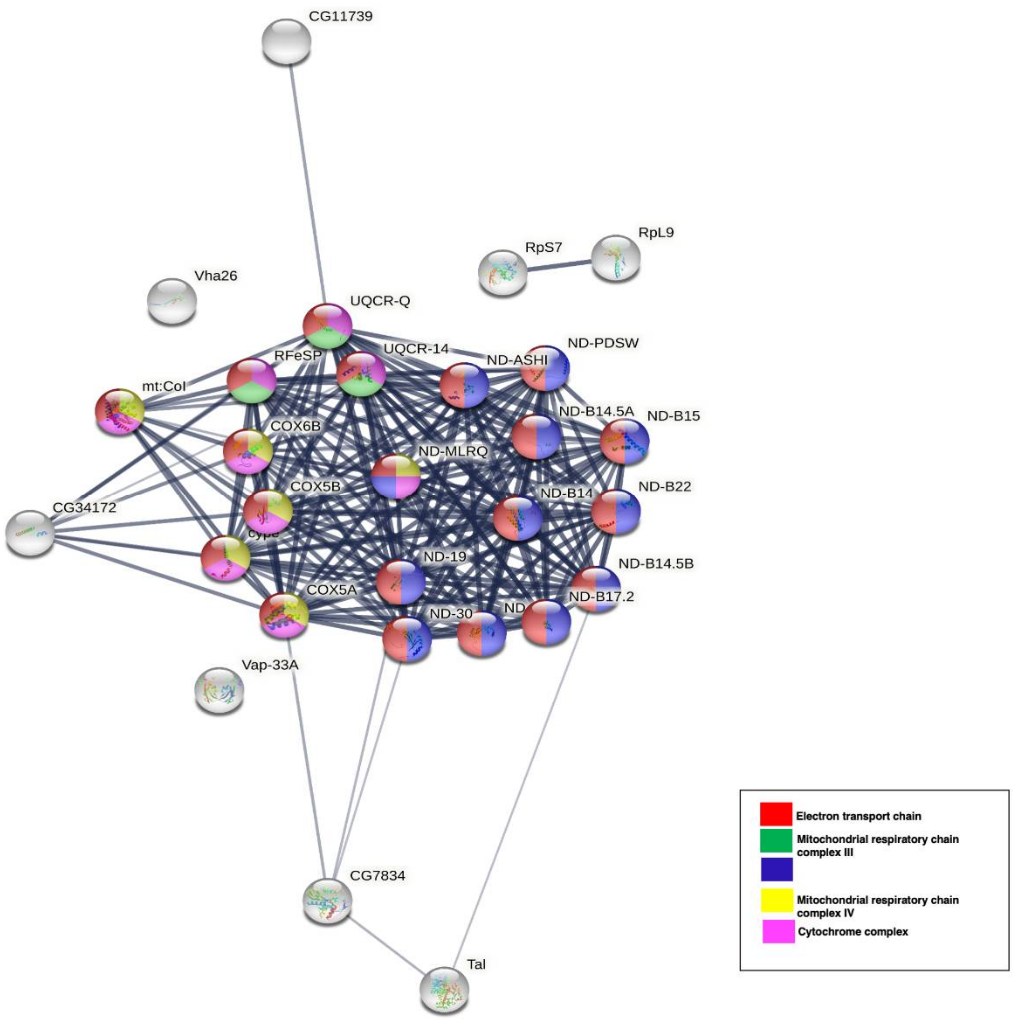

Supplementary Figure S1 - PPI network of mitochondrial proteins with increased abundance (RAR > 1.5) in response to one week of exercise in D. melanogaster. Network analysis by the STRING v11.0. software, with functional enrichment of (P < 1.0e-16).

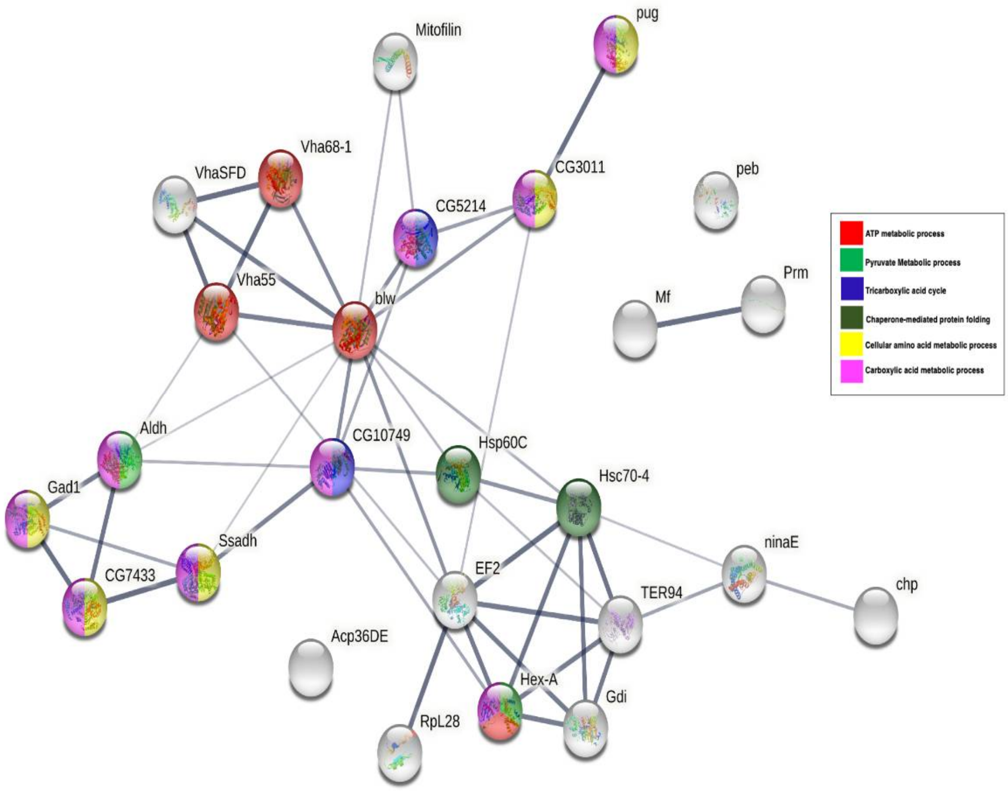

Supplementary Figure S2 – Protein-protein interaction network of mitochondrial proteins that have decreased abundance in response to one week of exercise in D. melanogaster. Network analysis by the STRING v.11.0. software, with functional enrichment of P < 2.75e-08.
